# A rapid review of challenges and opportunities related to diversity and inclusion as experienced by early and mid-career academics in the medicine, dentistry and health sciences fields

**DOI:** 10.1186/s12909-023-04252-x

**Published:** 2023-04-27

**Authors:** Marianne Piano, Kristin Diemer, Michelle Hall, Flora Hui, Elaina Kefalianos, Belinda J. Lawford, Gemma McKibbin, Rebecca J. Jarden

**Affiliations:** 1 Department of Optometry and Vision Sciences, Melbourne School of Health Sciences, University of Melbourne, 200 Berkeley Street, Carlton, VIC 3053 Australia; 2 National Vision Research Institute, Australian College of Optometry, Melbourne, Australia; 3 Department of Social Work, Melbourne School of Health Sciences, University of Melbourne, Melbourne, Australia; 4 Department of Physiotherapy, Melbourne School of Health Sciences, University of Melbourne, Melbourne, Australia; 5 Centre for Health, Exercise and Sports Medicine, University of Melbourne, Melbourne, Australia; 6 Centre for Eye Research Australia, Department of Surgery, Royal Victorian Eye and Ear Hospital, University of Melbourne, Melbourne, Australia; 7 Department of Audiology and Speech Pathology, Melbourne School of Health Sciences, University of Melbourne, Melbourne, Australia; 8 Department of Nursing, Melbourne School of Health Sciences, University of Melbourne, Melbourne, Australia; 9 Austin Health, Melbourne, Australia

**Keywords:** Academics, Diversity, Early Career, Inclusion, Mid-career, STEMM

## Abstract

**Background:**

Early- and mid-career academics in medicine, dentistry and health sciences are integral to research, education and advancement of clinical professions, yet experience significant illbeing, high attrition and limited advancement opportunities.

**Objectives:**

Identify and synthesise published research investigating challenges and opportunities related to diversity and inclusion, as experienced by early and mid-career academics employed in medicine, dentistry and health sciences disciplines.

**Design:**

Rapid review.

**Data sources:**

OVID Medline, Embase, APA PsycInfo, CINAHL and Scopus.

**Methods:**

We systematically searched for peer reviewed published articles within the last five years, investigating challenges and opportunities related to diversity and inclusion, as experienced by early and mid-career academics employed in medicine, dentistry and health sciences. We screened and appraised articles, then extracted and synthesised data.

**Results:**

Database searches identified 1162 articles, 11 met inclusion criteria. Studies varied in quality, primarily reporting concepts encompassed by professional identity. There were limited findings relating to social identity, with sexual orientation and disability being a particularly notable absence, and few findings relating to inclusion. Job insecurity, limited opportunities for advancement or professional development, and a sense of being undervalued in the workplace were evident for these academics.

**Conclusions:**

Our review identified overlap between academic models of wellbeing and key opportunities to foster inclusion. Challenges to professional identity such as job insecurity can contribute to development of illbeing. Future interventions to improve wellbeing in academia for early- and mid-career academics in these fields should consider addressing their social and professional identity, and foster their inclusion within the academic community.

**Registration:**

Open Science Framework (10.17605/OSF.IO/SA4HX).

**Supplementary Information:**

The online version contains supplementary material available at 10.1186/s12909-023-04252-x.

## Key messages

- 1. Of the 11 included studies of early- and mid-career academics, most reported concepts encompassed by professional identity, few of the findings related to the topic of inclusion or social identity, with sexual orientation and disability being a particularly notable absence.
- 2. Job insecurity, limited opportunities for professional development, and a sense of being undervalued in the workplace were evident for these academics.
- 3. Systems-level targeted action is needed to strengthen inclusion, diversity and support opportunities for advancement among early and mid-career academics.

## Background

The early- and mid-career academic (EMCA) workforce has experienced unprecedented challenges during the COVID-19 pandemic [1, 2], compounding pre-existing psychological illbeing associated with job insecurity [3], high workload [3, 4], and diversity of experience, discrimination, and sexism [2, 5]. Recruiting, maintaining, sustaining, and retaining these essential professionals is dependent on a range of factors including excellent leadership, management, workplace culture, support, promotion, and work-life balance [4, 6–17].

Work wellbeing is increasingly forefront in local [2], national [18] and international [19] recommendations for promoting mental health. Interventions to reduce illbeing (e.g., stress, anxiety, depressed mood, burnout) and promote flourishing and thriving in workplaces, including academia, have seen exponential growth [18, 20–22]. The experience of establishing a career in academia following completion of a Doctor of Philosophy degree is characterised by significant challenges such as limited support to navigate career progression, a problematic/dysfunctional workplace culture and unsustainable workloads affecting a sense of belonging and inclusion, and experiences of unacceptable workplace behaviours such as discrimination or bullying [2, 3, 23].

The Inclusion@Work Index [24] defines diversity as all the ways people differ in terms of their social and professional identities. When we consider this in the context of the workplace, diversity refers to the different social and professional identities of people in an organisation, such as, ethnicity, culture, age, gender, education and profession. Inclusion in the workplace is when this diverse mix of people are “respected, connected, progressing and contributing” [24] (p.7) to the success of an organisation; an individual’s identity will influence their views and experiences of inclusion.

In addition to the known pressures facing the EMCA workforce, recent research has identified a disproportionate impact of the COVID-19 pandemic upon early and mid-career researchers. Some identified impacts were relevant to diversity and inclusion issues, such as the greater impacts noted for females with caring responsibilities, or those working with reduced hours, and the noted loss of career prospects due to disrupted track records [2, 25, 26].

There is a large body of literature focusing on widening participation and mitigating diversity and inclusion challenges faced by postgraduate students at universities [27, 28]. Less is known about the experiences of academic staff in the same space [29]. Previous survey research that included clinician researchers indicates they may experience unique support needs compared to other STEM academics [30]. Findings from reviews and commentaries focused on science, technology, engineering and mathematics (STEM) disciplines [31–33] or academia generally [3, 29] may not have the same relevance to medicine, dentistry and health sciences fields.

The aim of this review was to identify and explore experiences and perceptions of diversity and inclusion, as experienced by early and mid-career academics employed in medicine, dentistry and health science disciplines. Synthesising primary research on these topics will inform future strategies and research to address challenges, capitalise on opportunities, and highlight successful initiatives.

There were three review questions (RQ), focused on the topic of experiences and perceptions of diversity and inclusion in early and mid-career academics employed in medicine, dentistry and health sciences: RQ1) what are the *characteristics of studies* about this topic? RQ2) what *instruments* have been used to investigate this topic? and RQ3) what are the reported *experiences and perceptions* in the included studies?

## Methods

This rapid review [34] followed an *a priori* review protocol. The review protocol was not eligible for registration on the PROSPERO platform due to the focus on experiences of academics, and therefore was registered in the Open Science Framework (https://doi.org/10.17605/OSF.IO/SA4HX) on 20^th^ July 2022. Registration occurred after searches and data extraction but before analysis. Reporting followed the updated guideline for reporting systematic reviews [35].

### Search strategy

#### Information sources

To identify potentially relevant studies, the following electronic databases were searched on 9^th^ November 2021: OVID Medline/Embase, APA PsycInfo, CINAHL and Scopus. Rapid review methodology guidance [36] suggests limiting to a single database – CINAHL was included due to our population including allied health academics, while APA PsycInfo and Scopus were included to ensure social sciences literature was considered, as publications relating to diversity and inclusion in the chosen context may be submitted to these journals rather than medical journals.

#### Search terms

The search strategy (reported in full in Supplementary File 1) was developed from the research questions and based on population, concepts, and context. A research librarian reviewed and advised on the final implemented search strategy.

### Inclusion and exclusion criteria

Only primary research papers reported in English from January 2017 to September 2021 were included. Constraining searches to recent literature, e.g. last 5 years, is a strategy deployed in rapid reviews with constrained time frames [37], as in the case of this project, to manage scope and feasibility. A review of reviews was not possible for this topic as we were unable to identify any published systematic reviews focusing on, or presenting separate data, for this subgroup.

#### Population

Early and mid-career post-doctoral academics in medicine, dentistry and health sciences (e.g., biomedical science, nursing, optometry, physiotherapy, social work, audiology) were included. The definition of early and mid-career academic varies between universities, funders and countries. For example, the EURAXESS definition of an early-stage researcher encapsulates the PhD study period [38], whereas other funding bodies exclude the PhD period and focus on the years post-award [17, 39]. For teaching specialists and mid-career researchers, this becomes even less delineated [21, 40]. A previous Australian study demonstrated the importance of permitting self-definition of career stage, to bridge the gap between current university definitions and the increasingly diverse experiences of early career academics [41]. For this reason, we included any article that reported early or mid-career academics as participants, and we extracted and reported any definitions provided by article authors. Science, technology, engineering, and doctoral students were excluded due to the wide body of previous work focusing on graduate students and the difference in challenges experienced by this cohort.

#### Concepts

Articles reporting experiences, perceptions, themes of diversity and inclusion in early and mid-career academics employed in medicine, dentistry and health sciences. Diversity in the context of early and mid-career academic social and professional identity were included. Diversity was defined as: “…all the differences between people in how they identify in relation to social identity, that is their Aboriginal and/or Torres Strait Islander background, age, caring responsibilities, cultural background, disability, gender, faith/no religion, LGBTIQ + status, and social class, and their professional identity, that is their profession, education, work experiences, and organisational role.” [24]. Inclusion was defined as occurring when “…a diversity of people are respected, connected, progressing and contributing to organisational success.” [24].

#### Context

Articles focused on tertiary education setting including medicine, dentistry, health, and biomedical sciences were included.

### Screening

The Cochrane Rapid Reviews Methods Group recommends dual screening at least 20% of abstracts with a single reviewer for the remainder after conflicts are resolved [36]. However, due to resourcing constraints it was not possible to have a single reviewer, therefore dual screening and extraction was applied to all abstracts to ensure consistency. Title and abstract screening was conducted by 7 reviewers, full text review by 3 reviewers, and data extraction by 3 reviewers. Conflicts arising during screening and full text review were resolved by consensus from members of the research team involved at each phase.

### Assessment of methodological quality

The assessment of methodological quality was conducted by a single reviewer (MP) using a range of established quality assessment tools as appropriate to the study design, such as those from the Critical Appraisal Skills Programme (CASP) and the National Institute of Health. Quality ratings, where tools indicated them, and supporting statements for the choice of rating were reviewed by a second reviewer (RJ). The assessment was limited to the study methodology and outcomes relating specifically to diversity and inclusion topics. Regardless of any ratings of methodological quality, all studies proceeded to data extraction and findings from this quality assessment are reported separately from the synthesis of findings. This approach was adopted due to the variety of study designs included in the review.

### Data extraction, analysis and reporting

Data extraction was limited to study characteristics (RQ1), outcome measures (RQ2), and findings (RQ3) specifically related to diversity and inclusion topics. The data extraction form was developed in Covidence by two reviewers and piloted on three papers to refine before full double extraction was undertaken. The Covidence platform highlighted conflicts in extraction which were resolved by consensus from members of the research team involved in this phase. The extracted data were tabulated, synthesised, and reported narratively to address each of the review questions.

## Results

### Study identification, screening and selection

**Fig. 1:**
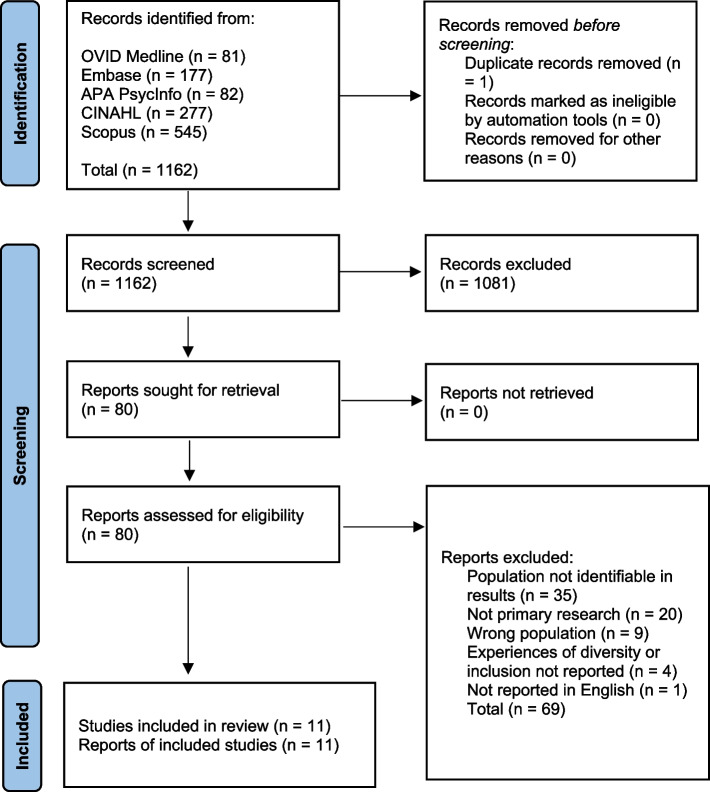
Study identification, screening and selection flowchart

Database searches were executed in September 2021, identifying 1162 articles; 11 met inclusion criteria, were quality appraised, and progressed to data extraction and analysis. A flowchart of study identification, screening and selection is illustrated in Fig. 1.

### Characteristics of included studies (RQ1 & RQ2)

Characteristics of included studies are reported in Table 1.

**Table 1:** Characteristics of included studies (*n* = 11)

Author	Country	Study Type	Population	Field	Study Aims	Intervention
Afonja, 2021 [42]	USA	Qualitative (survey)	*n* = 994, average time post-PhD 2.7 years (range not reported) F:M gender ratio: 3:2 13% underrepresented minorities (American Indian or Alaska Native, Black or African American, Native Hawaiian or other Pacific Islander, and/or Hispanic or Latino) Self-identify as postdoctoral scholar or research associate	Biomedical/ biological sciences	Identify the benefits, challenges, and strategies for pursuing an academic career	No intervention
Behar-Horenstein, 2017 [43]	USA	Qualitative (interview)	*n* = 5; all White F:M gender ratio: 3:2 Biomedical, behavioural and clinical research postdoctoral fellows funded by National Research Service Awards. ECR/MCR criteria not defined	Biomedical science, psychology and medicine	Describe mentee and mentor perspectives of how team science promotes collaboration and the development of independent researchers	T32 postdoctoral training program
Cameron 2020 [44]	USA	Longitudinal quantitative (survey)	*n* = 54 postdoctoral trainees across 33 states Mean age: 28 ± 4.5 years, range 22–46 years F:M gender ratio: 3:2 Self-identified ethnic groups: African American (12%), Asian/Asian American (23%), White (50%), Hispanic (13%), 76% native English speakers. 31% first-generation students ECR/MCR criteria not defined	Clinical science, biomedical science	Investigate development of scientific communication skills in role in academic research career intention	No intervention
Chaudron 2021 [45]	USA	Quantitative before and after study (intervention)	*n* = 49 All mid-career women researchers Definition: "mid-career women faculty, defined as associate professor or early professor"	Medicine, dentistry, nursing, biomedical science	Assess impact of a career development program on self-assessed knowledge, competence, self-identified goals, and promotions	6-month training program: -Small cohort approach to facilitate close relationships and networking -Informal networking event -4 days of activities to target developmental needs and assist in navigating leadership roles
Cumbler 2018 [46]	USA	Qualitative (interview)	*n* = 17 F:M gender ratio 1:1.12 Early career academic hospitalists self-identifying as clinician-educators from 3 academic medical centers. Average career duration 3.1 years, 94% were assistant professors, 12% were underrepresented minorities. ECR/MCR criteria not defined	Medicine	Explore barriers and facilitators of career success as perceived by early career academic hospitalists	No intervention
Deane 2021 [47]	UK	Qualitative (survey)	*n* = 34 postdocs; Participants in Facebook peer support group for healthcare professionals (doctoral and postdoctoral) in research. ECR/MCR criteria not defined	Nursing and Allied Health	Evaluate online peer support group including engagement, experiences, and identifying future career challenges	“Healthcare Professionals in Research” Facebook peer support group
Duke, 2020 [48]	USA	Cross-sectional survey	*n* = 66; all assistant professors F:M gender ratio: 7:4	Medicine (paediatrics)	Examine relationships between institutional context and burnout qualities to identify opportunities to address faculty burnout	No intervention
Eisen, 2017 [49]	USA	Mixed methods case study (survey, focus groups)	*n* = 46 (survey); 177 program fellows: *n* = 75 underrepresented minorities including 50% African-American, over 70% women	Biomedical science	Evaluate a postdoctoral program in terms of career success of participants	T32 FIRST postdoctoral training program
Lange, 2019 [50]	Netherlands	Qualitative (interview)	*n* = 13 postdoctoral F:M gender ratio: 2.25:1 ECR/MCR criteria not defined	Nursing	Explore the experiences and perceptions of postdoctoral nurses working in research with leadership and career development	No intervention
Price, 2018 [51]	USA	Qualitative (interview)	*n* = 30; 1–7 years post-PhD Gender ratio F:M 1.42:1 20% Hispanic, 80% not Hispanic Neuroscience (23%), Molecular and cell biology (20%) Biochemistry/biophysics/bioengineering (23%), Immunology (10%), other (23%)	Biomedical science	To better understand how postdoc scientific identities influence career goals	No intervention
van Dongen, 2021 [52]	Netherlands	Longitudinal mixed methods (interviews, survey)	*n* = 12; 2–8 years post-PhD F:M gender ratio: 3:1	Nursing	Evaluate expectations, experiences, and perceived influence of leadership mentoring program on leadership and professional development, professional identity, and research productivity of postdoctoral nurses	2-year leadership mentoring program: -5 × 2-day workshops with field experts -Development of leadership plan -Individual mentoring -Visit to research centre abroad

*Abbreviations*: *ECR/MCR* Early-Career Researcher/Mid-Career Researcher, *F* Female, *M* Male, *PhD* Doctor of Philosophy, *T32 FIRST* Fellowships in Research and Science Teaching post-doctoral training (T32), *UK* United Kingdom, *USA* United States of America

Of the included studies, five were published before 2020, and four published in the last year (2021). Study designs were primarily qualitative (*n* = 7), using interview or survey methods to explore experiences. Two studies used mixed methods (quantitative survey with qualitative interviews/focus groups [49, 52]). Three studies used a survey methodology to measure purely quantitative outcomes for statistical analysis [44, 45, 48]. Three studies looked at outcomes over time, with the remainder cross-sectional in design. Studies were predominantly USA-based (*n* = 8) with the remainder conducted in Europe.

Definitions of early and/or mid-career academics ranged from describing time since completing PhD to specific academic ranks (and titles) of participant. Participant career stage varied across studies, with only two studies reporting range of years post-PhD for participants [51, 52], totalling 1–8 years. Three studies had majority assistant, associate or early professors as participants and could be considered to have a focus on mid-career researchers. Criteria to define an early or mid-career researcher were not reported for 5 studies, while two studies defined postdoctoral status by participation in USA-specific “T32 FIRST (Fellowships In Research and Science Teaching)” programme, a nationwide programme of funded, institution-based postdoctoral research training. One study allowed participation by self-identification of postdoctoral status [42].

### Assessment of methodological quality

The methodological quality of included studies is tabulated in Supplementary File 2. Three studies were exploratory [45, 49, 52], assessing the impact of an established program. These studies did not report a research question or pre-determine a sample size and opted for mainly descriptive reporting. Most studies utilised participants within a single institution or were small sample, limiting transferability of findings to other program and institutions. Methods reporting was usually comprehensive. Only a limited proportion of the qualitative studies considered reflexivity. Some programs for early and mid-career academics were only accessible through a competitive selection process, therefore some biases cannot be ruled out when considering findings, such as candidates self-selecting based upon the criteria, and unconscious bias on part of those making the selection, potentially reinforcing traditional and conservative measures of performance. Many identified studies were US-based and may not necessarily generalise to other tertiary education systems in other countries.

### Experiences and perceptions (RQ3)

Findings related to diversity and inclusion were primarily focused on professional identity, and to a lesser extent, social identity. Main findings are reported in Table 2.

**Table 2:** Main findings related to diversity and inclusion

Author	Outcomes	Findings related to diversity and inclusion topics	
		**Social Identity (e.g., gender, ethnicity, sexual orientation, disability, caring responsibilities)**	**Professional Identity And Inclusion (e.g., profession, career progression, work culture)**
Afonja, 2021 [42]	Advice given to someone thinking about an academic research career	-Time and commitment required for success in academia affects familial obligations -Low salaries make it difficult to start a family	-Demanding workload (work-life balance important) -Lack of recognition for work as ECR: feeling undervalued -Limited opportunities for advancement -Lack of financial stability as ECR -Underpaid as ECR -Important to seek strong mentorship as ECRs -Building connections and networks is important for ECRs
Behar-Horenstein, 2017 [43]	Experiences of mentoring and team science	Nil	-Mentoring can be challenging if senior mentors advocate for their own research agenda -Mentoring resources helped ECRs: training seminars, conferences, access to patients, training in grant writing skills, and dedicated use of particular equipment or methodology -Team science approach to postdoctoral training programs enables access to multiple mentors, addressing issues with single mentorship such as conflicting perceptions of goals/abilities that may result in lack of benefit -Evolving emphasis on multidisciplinary translational research is a relatively new concept to academic departments and mentorship models need to evolve to match -Competition culture can strain relationships with peer mentors within faculty
Cameron 2020 [44]	Structural model to determine role of scientific communication in predicting science identity and research career intention	-Compared with native speakers, non-native speakers of English reported higher writing productivity and higher intention to become an independent investigator -Male trainees reported higher self-efficacy in informal speaking and lower intention to support science (not conduct research) -Trainees of under-represented groups reported higher outcome expectations than did trainees of well-represented groups Demographic variables (i.e., gender, rank, and language status) correlated with at least one intention indicator did not significantly predict the other career intention indicators	-Engaging in science communication and receiving active mentoring in science communication skills play a significant role in intention to persist in academic research careers at the postdoctoral level -Attending to and encouraging trainees to engage more in all forms of communication can help to strengthen their science identity and their intentions to persist in research
Chaudron 2021 [45]	Pre vs post-program self-assessed knowledge and competence Meeting of self-identified goals Promotions	Nil	Statistically significant improvements in knowledge and competency in all domains: promotional, organizational infrastructure, and communication 148 individual goals grouped into career development (32%), leadership (26%), networking (22%), negotiation (20%) – majority achieved e.g., promotion, grants, publications, honours/awards, committee memberships, new leadership roles) 23.5% of women were promoted or received tenure at last review
Cumbler 2018 [46]	Perceived barriers and facilitators for career success	Nil	Barriers: -Lack of protected non-clinical time to devote to academic pursuits -Burnout due to excessive work stress -Competing professional responsibilities Facilitators via Hospital Medicine Groups: -Support could be facilitated through individual mentorship and access to collegial networks -Time supporting work-life balance
Deane 2021 [47]	Experiences and perspectives about clinical academic career pathway	Nil	-12% cited lack of support in research and clinical environment -Need for a clearer clinical academic pathway -Concern about lack of career and job security -Isolation in clinical academic experience and lack of real-life support network; online group membership helped mitigate
Duke, 2020 [48]	Perceptions of value and empowerment felt within department 22-item Maslach Burnout Inventory (MBI)	Nil	Burnout Inventory: Odds of burnout in any dimension decreased with increasing perception of: -Ability to communicate professional needs, -Feeling valued for departmental contributions, -Departmental commitment to support faculty wellbeing Survey: -56% felt valued for their contributions to the department -52% felt empowered to communicate professional needs to colleagues and leadership -61% agreed that the department supported their overall wellbeing -11–15% met the criteria for burnout -Emotional exhaustion correlated with perceptions about institutional characteristics
Eisen, 2017 [49]	Diversity of participants, publication rate, employment, and attainment of career goals	-Program was inclusive of underrepresented minorities (URMs) (50% African American; 70% women) and these participants were as or more successful in their fellowships compared to other post docs, measured by publication rate, attainment of employment in academic science careers, and eventual research grant support	-Cohort-driven community and developmental teaching/research training were highly rated components of the program
Lange, 2019 [50]	Experiences and perceptions with leadership, career development, research, research productivity, workload, mentoring, and health	-Men were more vocal about their career ambitions and asked for career advancement -Women were more modest about their career ambitions, emphasizing the importance of improving care for patients	-Developing leadership and professional identity as a PhD nurse is a serious and conscious process -Desire to become a valuable member of clinical academia - Clinical academia perceived as an honourable but complex work environment -Continuous search for progression while balancing worlds (particularly when part-time between them) and tasks/commitments -New responsibilities came with fear of not being good enough; as time passed, confidence increased -Handling pressure of heavy workloads and high academic achievements was a huge challenge -Evening and weekend work necessary -Conflicts experienced between work and private life -Management, peer and family support highly important
Price, 2018 [51]	Discourses about life as a research scientist and barriers and facilitators to career progression	-Feeling that having children/family is not possible	-Increasing difficulty in obtaining grant funding as a major challenge to a PI’s academic freedom -Perceived poor work-life balance -Perceived long work hours -Feeling of failure if leave academy -Feeling of no plan B and ill-prepared to pursue other career outside of academia -Mentoring and professional development often doesn’t happen -Perceptions of only having a low salary -Temporary nature of position
van Dongen, 2021 [52]	Expectations, experiences, and perceived influence of program on leadership and development	Nil	Program perceived to be valuable, led to strengthened leadership skills, & development of professional identity. Participants demonstrated increased research productivity and promotions

With regard to social identity, only one study reported whether there were any transgender participants [51], and proportion speaking English as a second language [44]. Five studies reported ethnicity [42, 44, 49, 51], or race [46], while all but except Deane and Clunie [47] reported gender, with Chaudron et al.’s study [45] featuring all women participants. Five studies reported age [43, 44, 50–52]. Only two studies [51, 52] reported demographic information relating to marital status and dependents (i.e., children). None of the studies reported on living with disability or long-term health conditions.

Aspects of social identity were reported in five studies [42, 44, 49–51] including the topics of gender, family life, balance and children, ambition and success, and self-efficacy. Two studies reported differences in communication styles for males; finding males more vocal of career intentions [50] and more likely to perceive themselves as having high self-efficacy in informal speaking [44]. Academia impacted on having a family [51], as well as the amount of time spent with family [42]. All 11 studies reported findings related to professional identity. The main professional identity foci were relationships and connection, feeling valued and supported, and balancing clinical and research commitments.

Five studies reported interventions, comprising professional development [45, 49, 52], mentoring [43, 49, 52], or peer support/networking [45, 47]. Impact of these interventions was primarily reported positively, such as perceptions of enhanced development of professional identity and leadership skills, achieving set goals [45]. The USA-specific “T32 FIRST” postdoctoral research training program content was more tailored towards individuals, for example mentoring and having opportunities to work in an established laboratory [43, 49], although reported details of leadership education were limited. Those receiving mentoring found mentoring impactful upon their leadership skills, knowledge and competency [43, 44, 52]. Not all focused on these as specific outcome measures, for example, van Dongen et al. [52] considered research productivity as a measurable outcome. This was also the only intervention study to measure outcomes in a longitudinal manner.

From an inclusion perspective, findings from four studies [42, 47, 49, 50] emphasised value of feeling part of a community, for example as part of an intervention cohort, or peer support, or good line management, good connections, and networks. Online community supported this in lieu of an actual physical community [47]. Most findings related to inclusion explored experiences and perceptions [42, 43, 46–48, 50–52]. Studies reported challenges of heavy workloads [42, 46, 50], maintaining work-life balance [42, 46, 50, 51], imposter syndrome, and a lack of feeling supported and valued [42, 47, 48, 50], such as work not being appreciated by others [42]. A study by Duke et al. [48] exploring burnout in more depth, found the odds of experiencing burnout decreased if it was felt one’s contributions to a department were valued, and yet only half of participants felt their contributions were valued [48]. The authors did not speculate on reasons for this, instead highlighting steps taken at their institution to address these issues, such as creating opportunities to highlight faculty accomplishments, although the impact of these changes was not reported.

The lack of career support was particularly evident in the research and clinical environment for healthcare professionals [47] with challenges between wanting to progress but balancing roles as a clinician and a researcher [47, 50]. The complexity of heavy workloads in combination with high academic achievement [50] was also expressed as a warning to academics [42], suggesting work–life balance as essential. This striving for high academic achievement was also reported by Behar-Horenstein and Prikhidko [43], where the competition culture was felt to strain peer relationships. Opportunities for team science and multiple mentors were found to be beneficial [43], alongside relationships and connections in the form of networks [42, 49]. The included studies rarely had a direct focus on diversity and inclusion concepts, and subsequently did not go beyond reporting findings to interpret them and develop new concepts or theories. One exception was a study which examined mentoring experiences through the lens of team science [43], to understand the mechanisms by which mentoring may be beneficial.

In summary, the included studies largely focused on reporting a variety of experiences and perceptions relating to academic careers or participating in interventions to develop careers, a number of which related to diversity (social and professional identity) and inclusion.

## Discussion

Our rapid review aimed to synthesise the experiences and perceptions of diversity and inclusion by early and mid-career academics employed in medicine, dentistry and health sciences, as reported in the primary research literature. We have reported these experiences and perceptions as a narrative synthesis, alongside the characteristics of these studies, including the methods by which these studies have explored this topic.

There were limited findings in the reviewed studies relating to social identity, with disability and LGBTQIA + being particularly notable absences. Included studies primarily focused on ethnicity, race, and gender. LGBTQIA + status was rarely reported in these studies, and we found no studies where exploring this was a specific aim. One paper incidentally reported transgender status alongside male and female genders [51]. By contrast, there are studies that have explored LGBTQIA + discrimination in STEM more generally, or from the student experience perspective [53, 54], with similar papers existing for students with disabilities [55–57]. None of the included studies focused on disability. It appears there is limited focus on certain aspects of social identity in both the EMCA space and overall fields of medicine, dentistry and health sciences.

Included studies frequently addressed concepts encompassed by professional identity, often echoing those in papers about other areas of academia [6, 7, 30], but very few of the findings relate to the topic of inclusion. These studies used data collected before COVID-19 introduced novel challenges to academia, such as physical distancing, altered education pathways and moving of programmes online. Surveys of EMCAs during the pandemic highlight the challenges of creating a sense of belonging and inclusion under such circumstances, e.g. feeling connected to colleagues, as well as opportunities to network and be involved in the workplace culture [2, 25, 26]. In clinical academia, COVID-19 also compounded challenges related to professional identity, such as high workloads, and work-life imbalance, and added to psychological illbeing, such as high stress and anxiety [20, 45, 51]. These impacts are also further accentuated by the social identity of EMCAs, such as feeling unable to start a family due to lack of job security or low pay, feeling unable to meet current family obligations, or having other caring responsibilities necessitating, for example, part-time working [2, 25, 26, 42, 51]. Overall, there are a number of work-based contributors to poor mental health for EMCAs that are inextricably linked to diversity and inclusion concepts.

Work plays an important role in mental health, and is forefront in the World Health Organization’s definition of mental health as “a state of wellbeing in which the individual realises his or her own abilities, can cope with the normal stresses of life, can work productively and fruitfully, and is able to make a contribution to his or her community” [58]. When considering findings related to inclusion, there are several similarities with models of workplace wellbeing developed through academic research [59] which typically include positive relationships and emotions, life purpose/meaning, personal growth, autonomy, engagement and self-acceptance. These components resonate with findings from studies in our review that demonstrate the value of mentoring relationships, a sense of community, and networking opportunities or interventions supporting the meeting of personal goals, or development of leadership skills. What is not evident in academic conceptualisations of workplace wellbeing [59], yet is evident in our review, was the importance of academics feeling supported, respected, and valued. It can therefore be argued that interventions to improve diversity and inclusion experiences of early and mid-career academics in the higher education space, with a particular focus on the less explored areas medicine, dentistry and health sciences, would be highly relevant to improve work wellbeing, and subsequently mental health.

With regard to interventions, we found a limited number for early and mid-career academics in medicine, dentistry and health sciences fields that reported an accompanying evaluation – papers that described programs but did not report outcomes were excluded. Intervention studies had varied aims and objectives but often sought to establish a cohort or community [43, 49], and thus a sense of belonging and inclusion. However, experiences and perceptions relating to diversity and inclusion were a ‘by-product’ of the research process, and the programmes described did not appear to have fostering inclusion as a primary aim, despite many reporting social identity characteristics. There was also no theoretical framework described for the foundations of these interventions, although one study retrospectively explored the “T32 FIRST” postdoctoral research training programme in the context of the team science framework [43]. Many interventions were limited in their description within the reviewed papers, for example the components of leadership programmes were not described in depth.

A future opportunity is to consider a systematic approach to the development, implementation and evaluation of programs to actively promote wellbeing of academics and enhance inclusivity. Interventions to enhance wellbeing could be informed by recognised academic models of wellbeing, such as the “Five Ways to Wellbeing” [60, 61], which includes: ‘Connect’, ‘Be active’, ‘Take notice’, ‘Keep learning’, and ‘Give’. Use of a systematic reporting template, appropriate to the intervention, may strengthen reporting of these interventions, in turn supporting their future reliable implementation and replication. One such example is the *Template for Intervention Description and Replication* (TIDieR [62]). Unifying definitions of diversity and inclusion, determining the best evidence to inform the development of these interventions, and accurately measuring the effectiveness of interventions are all crucial next steps.

A key point of interest in these intervention studies was the variety of definitions (or lack thereof) of early and mid-career researcher used to determine access to these interventions. In some countries membership of post-doctoral associations is simply by self-definition of being an EMCA. It is important to consider, from an inclusion perspective, whether the lack of consistent definition of what constitutes an early or mid-career researcher may in fact be a barrier to some individuals putting themselves forward for these programmes [40]. Further, unifying definitions globally would help to determine the transferability of some of these interventions to other locations.

Based on our review findings, including lack of clarity in definitions of EMCAs and limitations of the available evidence, we recommend strengthening the evidence base to inform future interventions and diversity and inclusion experiences of EMCAs through the development of:

1. Global unified definitions of early career academic and mid-career academics.
2. A planned, global approach to exploration and description of diversity and inclusion research for early- and mid-career academics.
3. A planned, global approach to development and evaluation of interventions to improve diversity and inclusion experiences of early- and mid-career academics.

### Limitations

Resourcing constraints necessitated some deviations from the Cochrane recommendations for rapid reviews, such as the use of multiple reviewers across different screening phases and data extraction. The impact of this was mitigated by applying dual screening, resolution of screening conflicts with the research team and double extraction of the included articles. The variation in definitions of early and mid-career academic across studies has been explicitly described in the tables but applying the findings of such studies to other countries with different definitions is more difficult. Similarly, there may be differences between countries in the way some groups of academics captured within our target population are treated, such as differing pay rates for clinical academics versus clinical practitioners [63], or clinical versus non-clinical academics. These differences could affect experiences and perceptions of certain topics such as job security and financial stability to support family.

The search terms and chosen databases are likely to bias towards research conducted in English-speaking countries, and will not capture, for example, project reports on websites for higher education organisations. While we endeavoured to capture a variety of search terms that describe early to mid-career academics, it’s possible that some articles may have been missed if the cohort was described in a way that did not refer directly to this career stage in the title or abstract. We have clearly reported the constrained timeframe of searches to the last 5 years of published primary research so the scope of our review is clear, thus older literature is not represented. Lastly, although data screening and extraction were performed in groups, we cannot rule out our respective social and professional identities impacting upon the narrative interpretation of the literature (see authors’ information).

## Conclusions

Our review has identified that academic models of wellbeing overlap with key facilitators of inclusion, and key aspects of professional identity such as job insecurity can contribute to the development of illbeing. As such, wellbeing, illbeing, diversity and inclusion are linked. Therefore, to improve wellbeing in academia for early- and mid-career academics, future interventions, particularly in the relatively under-researched fields of medicine, dentistry and health sciences, should consider addressing their social and professional identity, and how they are included within the academic community. There is an opportunity to strengthen primary research on academic wellbeing through the inclusion of groups with certain social identities not currently represented within this research area, such as people who identify as LGBTQIA + and people with disabilities, who are absent from recent literature.

## Supplementary Information

**Additional file 1: Supplementary file 1.** Full search strategy. **Supplementary file 2.** Methodological quality of included studies.

## Data Availability

All data generated or analysed during this study are included in this published article and supplementary information file. An example search string using the CINAHL database is archived at searchrxiv: https://doi.org/10.1079/searchRxiv.2022.00083.
