# Supplementary material for: A rapid review of challenges and opportunities related to diversity and inclusion as experienced by early and mid-career academics in the medicine, dentistry and health sciences fields
Source: BMC Med Educ. 2023 Apr 27;23:288. doi: 10.1186/s12909-023-04252-x (PMC10139666; doi:10.1186/s12909-023-04252-x)
Supplement: Supplementary file 1 — Additional file 1: Supplementary file 1. Full search strategy. Supplementary file 2. Methodological quality of included studies. [file 12909_2023_4252_MOESM1_ESM.pdf]

## Supplementary files

### Supplementary file 1. Full search strategy

The terms used for the CINAHL database are shown below as an example.

( TI ( ((early-career OR mid-career OR junior) N2 researcher*)) OR ((early-career OR mid-career OR junior) N2 academic*)) OR ((early-career OR mid-career OR junior) N2 lecturer)) OR (post-doc* OR postdoc* OR post-PhD) OR (teaching and fellow) or (ECR or ECRs or MCR or MCRs or EMCR or EMCRs) ) OR AB ( ((early-career OR mid-career OR junior) N2 researcher*)) OR ((early-career OR mid-career OR junior) N2 academic*)) OR ((early-career OR mid-career OR junior) N2 lecturer)) OR (post-doc* OR postdoc* OR post-PhD) OR (teaching and fellow) or (ECR or ECRs or MCR or MCRs or EMCR or EMCRs) ) ) AND ( MJ ( Education OR Education, Dental OR Education, Medical OR Education, Nursing OR Health Education OR Dental Health Education OR Education, Premedical OR Education, Nursing, Research-Based OR Research, Nursing OR Research OR Health Services Research OR Research Personnel OR Medicine OR Dentistry OR Nursing as a Profession OR Allied Health Professions OR Allied Health Personnel OR Research, Medical ) OR TI ( research* or educat* or institut* or universit* or academi* ) OR AB ( research* or educat* or institut* or universit* or academi* ) ) ) AND ( ( ( MJ ( Cultural Diversity OR Culture OR Prejudice OR Ageism OR Gender Equality OR Homophobia OR Racism OR Sexism OR Weight Bias OR Disabled OR Health Status OR Mental Health OR Social Norms OR Gender Role OR Minority Groups OR Demography OR Ethnic Groups OR Health Status OR Sexism OR Parenting or Parents or Parent-Child Relations or Mothers or Pregnancy or Fathers or Family or Child ) OR ( TI ( diversity or social identity or parent* or father* or pregnan* or mother* or mum* ) OR AB ( diversity or social identity or parent* or father* or pregnan* or mother* or mum* ) ) ) OR ( MJ ( Career Planning and Development OR Career Mobility OR Leadership OR Mentorship OR Morale OR Demoralization OR Role OR Professional Role OR Organizational Culture ) OR TI ( mentor* promot* or advoca* or success* or funding or career* or progress* or develop* or secur* or diversity or professional identity ) OR AU ( mentor* promot* or advoca* or success* or funding or career* or progress* or develop* or secur* or diversity or professional identity ) ) OR ( MJ ( Social Inclusion OR Interprofessional Relations OR Peer Group OR Peer Review OR Research Support ) OR TI ( belonging OR inclusion OR culture ) OR AB ( belonging OR inclusion OR culture ) ) ) )

Published Date: 20170101-20211231

Supplementary file **2. Methodological quality of included studies.**

| | Tool | Q1 | Q2 | Q3 | Q4 | Q5 | Q6 | Q7 | Q8 | Q9 | Q10 | Q11 | Q12 | Q13 | Q14 | Additional appraisal tool notes |
|---|---|---|---|---|---|---|---|---|---|---|---|---|---|---|---|---|
| Afonja (2021) | CASP - Q | Y | Y | Y | Y | Y | N | Y | Y | Y | OK | | | | | |
| Behar (2017) | CASP - Q | Y | Y | Y | Y | Y | Y | Y | Y | Y | OK | | | | | |
| Cameron (2020) | NIH – X | Y | Y | N | Y | N | N/A | N/A | N/A | Y | N/A | Y | N/A | Y | Y | Fair |
| Chaudron (2021) | NIH – P/P | Y | Y | Y | N | Y | Y | Y | N | N | Y | N | N | | | Fair |
| Cumbler (2018) | CASP - Q | Y | Y | Y | Y | Y | N | Y | Y | Y | LIMITED | | | | | |
| Deane (2021) | CASP - Q | Y | Y | Y | Y | Y | Y | Y | Y | Y | OK | | | | | |
| Duke (2020) | NIH – X | N | Y | Y | Y | N | N/A | N/A | N/A | N/A | N/A | Y | N/A | N/A | Y | Fair |
| Eisen (2017) | MMAT | N | N | | | | | | | | | | | | | No further questions responded as research question unclear |
| Lang (2019) | CASP - Q | Y | Y | Y | Y | Y | N | Y | Y | Y | OK | | | | | |
| Price (2018) | CASP - Q | Y | Y | Y | Y | Y | N | Y | Y | Y | OK | | | | | |
| VanDongen (2021) | MMAT | N | N | | | | | | | | | | | | | No further questions responded as research question unclear |

*Abbreviations*: CASP – Q = Critical Appraisal Skills Programme – qualitative study appraisal tool (https://casp-uk.net/casp-tools-checklists/); MMAT = Mixed Methods Appraisal Tool (MMAT) Version 2018 (http://mixedmethodsappraisaltoolpublic.pbworks.com/w/file/fetch/127916259/MMAT_2018_criteria-manual_2018-08-01_ENG.pdf); NIH – X = National Heart, Lung, and Blood Institute Quality Assessment Tool for Observational Cohort and Cross-Sectional Studies (https://www.nhlbi.nih.gov/health-topics/study-quality-assessment-tools); NIH – P/P = National Heart, Lung, and Blood Institute Quality Assessment Tool for Before-After (Pre-Post) Studies With No Control Group (https://www.nhlbi.nih.gov/health-topics/study-quality-assessment-tools).
